# Supplementary material for: Variation of miRNA Content in Cow Raw Milk Depending on the Dairy Production System
Source: Int J Mol Sci. 2022 Oct 2;23(19):11681. doi: 10.3390/ijms231911681 (PMC9569736; doi:10.3390/ijms231911681)
Supplement: Supplementary file 1 [file ijms-23-11681-s001.zip › ijms-1905990-supplementary.pdf]

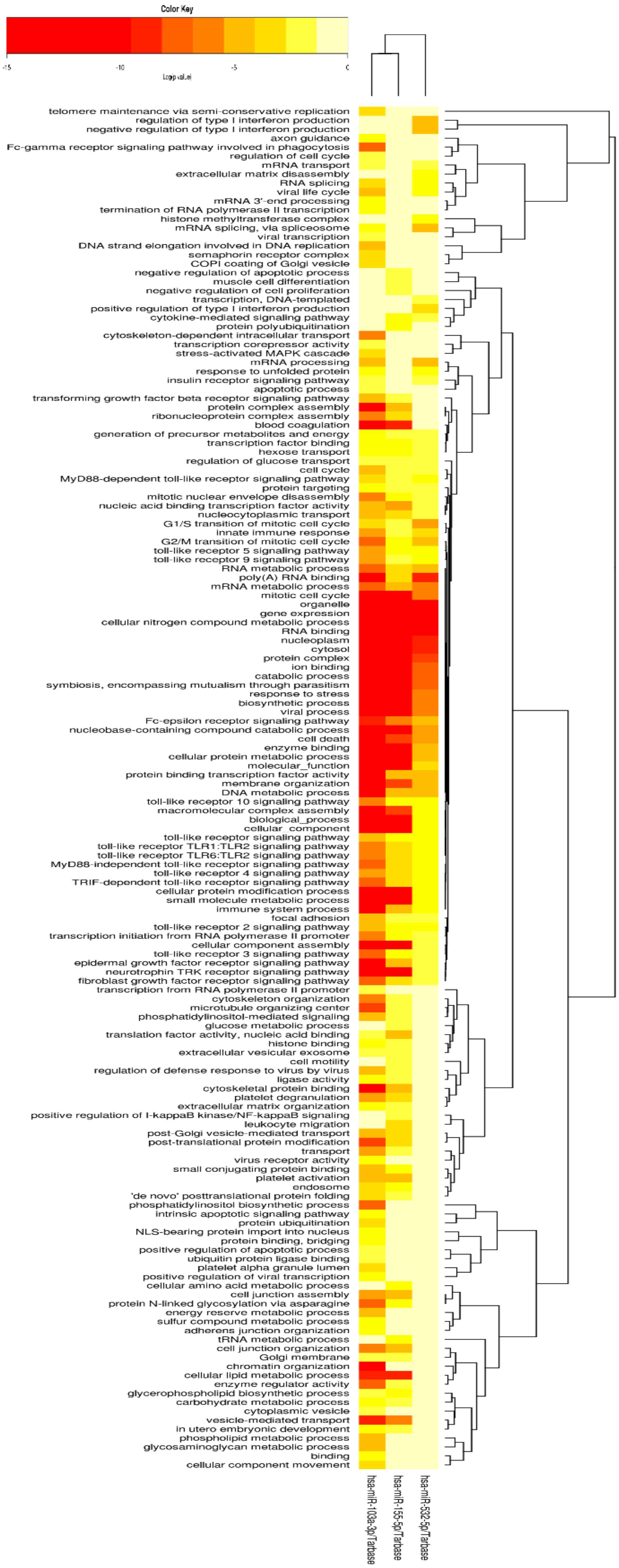

Supplementary figure S1. Heatmap of hierarchical clustering of *miR-103, miR-155* and *miR-532* based on mRNA target pathways, identified in DIANA using the Tarbase and GO intersection representation. Darker colors represent lower p-values.
